# Electromyographic Activity of the Shoulder Muscles During Arm Elevation in Asymptomatic Subjects—A Cross-Sectional Study

**DOI:** 10.3390/jfmk11020161

**Published:** 2026-04-21

**Authors:** Martin E. Barra-López, Carlos López-de-Celis, Erik Garcia-Ribell, Sergi Rodríguez-Rodríguez, Miguel Malo-Urriés, Jacobo Rodríguez-Sanz

**Affiliations:** 1 Department of Physiotherapy, Faculty of Medicine and Health Sciences, Universitat Internacional de Catalunya, Carrer de Josep Trueta s/n, 08195 Sant Cugat del Vallès, Spain; 2 Study Group on Pathology of the Locomotor System in Primary Care (GEPALAP), Institut Universitari d’Investigació en Atenció Primària (IDIAP Jordi Gol), Gran Via de les Corts Catalanes, 587, 08007 Barcelona, Spain; 3 ACTIUM Functional Anatomy Group, Faculty of Medicine and Health Sciences, Universitat Internacional de Catalunya, Carrer de Josep Trueta s/n, 08195 Sant Cugat del Vallès, Spain; erik.garcia@uic.es (E.G.-R.); srodriguezr@uic.es (S.R.-R.); jrodriguezs@uic.es (J.R.-S.); 4 Department of Medicine, Faculty of Medicine and Health Sciences, Universitat Internacional de Catalunya, Carrer de Josep Trueta s/n, 08195 Sant Cugat del Vallès, Spain; 5 Department of Physiatry and Nursing, Health Sciences Faculty, University of Zaragoza, Calle de Domingo Miral s/n, 50009 Zaragoza, Spain; malom@unizar.es; 6 PhysiUZerapy Health Sciences Research Group, University of Zaragoza, Calle de Domingo Miral s/n, 50009 Zaragoza, Spain

**Keywords:** shoulder, electromyography, natural movements, sex differences

## Abstract

**Background:** Although several studies have compared muscle activity in ‘healthy’ and ‘unhealthy’ shoulders, studying ‘healthy’ shoulders alone could improve the understanding of shoulder biomechanics. **Objective:** This study aims to describe the electromyographic activity of several shoulder muscles during a full range of free active flexion, as well as during abduction and scaption movements, and to compare gender differences in subjects with no history of shoulder pain or pathology. **Methods:** A cross-sectional descriptive study was conducted with 34 subjects aged between 18 and 60 years of both genders. The activity of the anterior, middle, and posterior deltoid, serratus anterior, infraspinatus, latissimus dorsi, and teres major muscles was measured using surface electromyography. Root Mean Square (RMS) values were calculated as a percentage of Maximal Voluntary Isometric Contraction (MVIC). **Results:** Regardless of whether they are considered agonists or antagonists, these muscles were active, with no statistically significant differences (Mann–Whitney *U* test), during both the lifting and lowering phases of the studied movements. Statistically significant differences between movements were observed only in the deltoid (Kruskal–Wallis *H* test, *p* < 0.004), which was more active during abduction. Women showed statistically significant muscle activity increase compared with men in some movements, except in the infraspinatus muscle—for example, in the three parts of the deltoid during the lifting phase of scaption (ANCOVA, *p* = 0.002–0.024). **Conclusions:** In this sample, the shoulder muscles studied showed comparable activity, acting as agonists or antagonists during shoulder elevation. These findings are exploratory and may help inform future studies on muscle activation in healthy shoulders during more varied functional tasks.

## 1. Introduction

Elevation of the upper limb is the movement with the greatest range of motion in the human body. It moves the arm away from the trunk in all directions and, together with elbow flexion and extension, determines the area of action of the hand during most activities of daily living [1].

The natural gesture of raising the upper limb does not correspond to the anatomical movements of flexion or abduction, which are defined with respect to the sagittal and frontal anatomical planes, respectively. As early as the 1930s, some authors advocated using the scapular plane as a standard reference when describing shoulder movements [2]. The acronym scaption was coined to label the elevation movement performed in a more natural and functional plane. It is interpreted interchangeably as ‘scapular plane abduction’ [3] or ‘scapular plane elevation’ [4].

The so-called ‘scapulohumeral rhythm’ [5], which is the coordinated muscular activity across the scapulohumeral and scapulothoracic joints, is critical for achieving overhead positions and ensuring optimal shoulder function [6]. Conversely, poor neuromuscular control and altered shoulder muscle activity are major contributing factors to a variety of shoulder conditions [7,8,9].

Electromyography (EMG) is a useful tool for studying muscle activity because it provides information about muscle activation throughout the full range of motion [10]. Several studies have investigated electromyographic muscle activity during various rehabilitation exercises [11,12,13], in specific pathological contexts [14], and regarding the role of the rotator cuff [15] and the scapulothoracic motion [16] in healthy individuals. Nevertheless, the available evidence on muscle activation patterns during the everyday use of the upper limbs remains limited, as relatively few studies have examined upper limb activity during activities of daily living [17,18]. Characterizing shoulder muscle activity during everyday movements could help establish a more ecologically valid framework for studying shoulder function.

The present cross-sectional descriptive study aimed to evaluate shoulder muscle activity in the dominant upper extremity using surface electromyography during unloaded, free active flexion, abduction and scaption movements across the full range of lifting and lowering phases. A further objective was to compare gender differences in a sample of asymptomatic subjects with no history of shoulder pain or pathology.

## 2. Materials and Methods

### 2.1. Study Design

A cross-sectional descriptive study was conducted in the research laboratory of Universitat Internacional de Catalunya. The study protocol (Study Code: FIS-2023-06) was approved by the local ethics committee of Universitat Internacional de Catalunya (CEIm—Comitè d’Ètica d’Investigacions amb Medicaments) on 17 October 2023. The study procedures were conducted in accordance with the Declaration of Helsinki: Ethical principles for medical research involving human subjects (64th General Assembly of the World Medical Association, Fortaleza, Brazil, October 2013). The participants were informed both verbally and in writing. The privacy rights of each participant were observed, and written informed consent was obtained from all of them.

### 2.2. Sample

Between September and October 2024, 34 volunteers from de Universitat Internacional de Catalunya community were recruited. Inclusion criteria were individuals aged between 18 and 60 years of both genders, asymptomatic, with no history of pathology or previous injury in the cervicoscapular or the shoulder region. Subjects over the age of 60 were not recruited due to the high probability of presenting asymptomatic injuries, including rotator cuff tears [19]. Participants were excluded if they met any of the following criteria: (1) range of motion <180° of elevation in the dominant upper extremity; (2) a diagnosis of systemic pathology such as diabetes or hypertension; (3) pharmacological medical treatment that could interfere with the measurements such as anticonvulsants and antidepressants; and (4) insufficient language skills to understand the informed consent or study instructions. Table 1 summarizes the demographic characteristics of the sample, including normalization data for the analyzed muscles.

### 2.3. Procedures

#### 2.3.1. Electromyography

The reliable and validated surface electromyography (sEMG) mDurance^®^ system (mDurance Solutions SL, Granada, Spain) was used to record muscle activity during a functional task (ICC = 0.916; 95% confidence interval [CI] = 0.831–0.958) [20]. The mDurance^®^ system consists of an EMG Shimmer3 unit (Realtime Technologies Ltd., Dublin, Ireland). This unit is a bipolar surface electromyography sensor for the acquisition of muscle activity. The common mode rejection ratio was 110 dB. Each Shimmer3 has two channels, with a sampling rate of 1024 Hz, applying a bandwidth of 8.4 Hz, and a 24-bit signal with an overall amplification of 100 to 10,000 *v*/*v* [20]. The mDurance Android app receives the data from the Shimmer and sends them to cloud service where the data are stored, filtered, and analyzed [20].

Raw EMG signals were first band-pass filtered using a fourth-order Butterworth filter (20–450 Hz). Then, the signals were rectified and smoothed using a moving RMS window of 0.025 s with 0.0125 s overlap [20]. The Maximal Voluntary Isometric Contraction (MVIC) was calculated using the RMS signal during isometric tests, the methodology for which is outlined below.

The main variable recorded for muscle activity was Root Mean Square (RMS) expressed as a percentage of Maximal Voluntary Isometric Contraction (MVIC) measured in microvolts (µV). A moving RMS smoothing filter was applied to the EMG signals, implemented with a 500 ms window (250 ms backward and 250 ms forward) for each signal sample. The signal was analyzed by the average [20].

#### 2.3.2. Study Procedure

After the subject had decided to participate in the study and their eligibility had been verified, they provided written informed consent. The investigators then proceeded to collect the following baseline characteristics: age, gender, weight, height, body mass index, and upper extremity dominance (Table 1).

Next, the subject’s skin was cleaned with alcohol and dried before the electrodes were placed. If hair impeded the correct adhesion of the electrodes to the skin, the site was shaved. Self-adhesive 5 cm Valutrode^®^ surface electrodes were placed on the muscle belly of the assessed muscles according to the SENIAM project [21] recommendations, with an interelectrode distance of 20 mm [22].

To normalize the data, a Maximal Voluntary Isometric Contraction (MVIC) was performed [23]. “Empty can” test position was used to test anterior, middle, and posterior deltoid and serratus anterior [23]. “External rotation 90°” test position was used to test the infraspinatus [23]. “Internal rotation 90°” test position was used to test the latissimus dorsi [23] and the teres major [24]. The MVIC of each muscle was obtained by averaging the peak of the RMS signal from three maximal contractions of five seconds against a fixed support (gym equipment adjusted to each participant), to ensure that maximal force was always isometric, with 30 s of recovery between each repetition. A 10-min rest period was granted after the normalization tests.

#### 2.3.3. Muscles and Movements Evaluated

The muscles assessed were the anterior deltoid, the middle deltoid, the posterior deltoid, the serratus anterior, the infraspinatus, the latissimus dorsi, and the teres major of the dominant upper limb. The method used to determine the dominant limb was self-report [25].

The selected muscles are: three shoulder abductors (the anterior, middle and posterior deltoids), three adductors (the infraspinatus, the latissimus dorsi and the teres major), three internal rotators (the anterior deltoid, the latissimus dorsi and the teres major), two external rotators (the posterior deltoid and the infraspinatus) and the serratus anterior as a scapular stabilizer.

Muscle activity was measured during the initial lifting and subsequent lowering phases of the full active range of motion in flexion and abduction in the sagittal and coronal anatomical planes, respectively, as well as in elevation (scaption) in the scapular plane.

**Figure 1:**
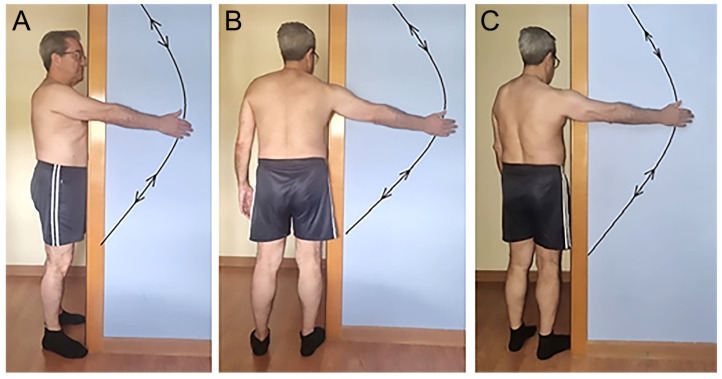
Position of the subjects to measure: (**A**) flexion; (**B**) abduction; and (**C**) scaption.

To ensure that the desired plane is not altered throughout the full range of the assessed movements, participants (Figure 1) were required to stand close to a door frame, with the torso positioned perpendicular to the wall (for flexion), parallel to the wall (for abduction) and at 35° to the wall (for scaption). The elbow was maintained in an extended position, and the palm of the hand, with the thumb pointing upwards (referred to as the “full can” position), was gently slid into contact with the wall during the measurement process, with no force being applied to the wall itself.

The subjects were required to execute several elevations of the upper extremity, to familiarize them with the electrodes and the procedure prior to the recording of muscle activation. During this training period, the participants were instructed to adopt a slow up-and-down rhythm of approximately three seconds, utilizing both visual and auditory stimuli as exemplars. The sequence of movements was always the same: flexion, abduction and scaption.

#### 2.3.4. Statistical Analysis

Statistical analysis was performed using the SPSS v.25 statistical software package. Descriptive statistics were performed for all the variables. The Shapiro–Wilk test was used to assess whether the data were normally distributed, and homogeneity of variances was verified prior to conducting the one-way ANOVA. To compare muscle activity between the lifting and lowering phases of each movement, the independent samples *t*-test was used when the data were found to be normally distributed; otherwise, the Mann–Whitney *U* test was used. Effect sizes were calculated using Cohen’s *d*. Values of 0.14, 0.31, and 0.61 were considered as small, medium, and large effect, respectively [26]. To compare muscle activity between movements, one-way ANOVA was used when the data were found to be normally distributed and the assumptions were reasonably met; otherwise, the Kruskal–Wallis *H* test was used. If significant differences were observed, Tukey or Dunn post hoc tests were applied with Bonferroni-adjusted significance levels to control the family-wise error rate across all pairwise comparisons. To compare muscle activity between sexes, analysis of covariance (ANCOVA) was employed, adjusting for anthropometric and Maximum Voluntary Isometric Contraction (MVIC) covariates when statistically significant baseline differences were observed. Effect sizes were calculated using eta squared (η^2^). An effect size >0.14 was considered as large, around 0.06 as medium, and <0.01 as small [27]. All statistical tests were performed at an alpha level of 0.05. When multiple pairwise comparisons were carried out (i.e., post hoc Tukey or Dunn tests), Bonferroni-adjusted *p*-values were used to control the family-wise error rate.

## 3. Results

**Figure 2:**
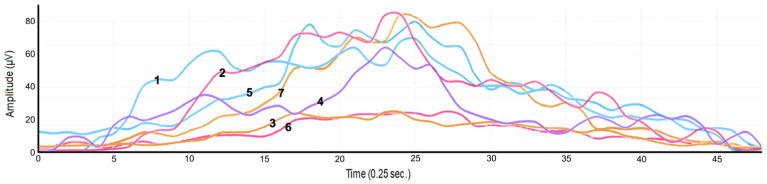
Electromyographic activity of a subject during flexion movement. (1) Anterior deltoid; (2) middle deltoid; (3) posterior deltoid; (4) serratus anterior; (5) infraspinatus; (6) latissimus dorsi; (7) teres major.

Table 2 shows the RMS values for the lifting and lowering phases of the analyzed movements. All the muscles under investigation were active during both the lifting and lowering phases of all movements. The serratus anterior demonstrates the greatest activity in all three analyzed movements both during lifting and lowering, revealing the pivotal role of scapular stabilization in arm movement. Electromyographic activity on the left shoulder of a subject during flexion movement (i.e., the lifting and lowering phases) is shown in Figure 2.

No statistically significant differences were observed between the two phases, regardless of whether the muscles are considered agonists or antagonists for that movement. However, a trend towards greater activation was observed during the phase involving concentric contractions. The serratus anterior, the anterior deltoid, the middle deltoid and the posterior deltoid are more active during the lifting phase. Conversely, the infraspinatus, the latissimus dorsi and the teres major are more active during the lowering phase, except for the latissimus dorsi during scaption.

With reference to the deltoid muscle, the anterior part is most active during flexion and scaption (RMS 20.41 and 16.41, respectively), while the middle part is most active during abduction (RMS 13.09). Abduction in the coronal plane requires a higher level of activity from the entire deltoid muscle group during both lifting (total RMS 37.1) and lowering (total RMS 32.16), compared to flexion (total RMS 32.95 during lifting and 28.60 during lowering) and scaption (total RMS 31.62 during lifting and 28.00 during lowering).

The latissimus dorsi and teres major muscles, both synergists with respect to the glenohumeral joint, show a high degree of similarity in their activation patterns during the analyzed movements. Nevertheless, during scaption, the latissimus dorsi was more active during lifting (RMS 12.04), while the teres major was more active during lowering (RMS 12.36).

It is widely acknowledged that external rotation plays a pivotal role in mitigating subacromial impingement during arm elevation [28]. Of the two external rotators included in this study, the posterior deltoid demonstrated greater activity during the lifting phase of all three analyzed movements, while the infraspinatus showed increased activity during the lowering phase (flexion RMS 8.52, abduction RMS 9.06 and scaption RMS 8.63), compared to the lifting phase (flexion RMS 8.01, abduction RMS 7.98 and scaption RMS 8.62).

**Figure 3:**
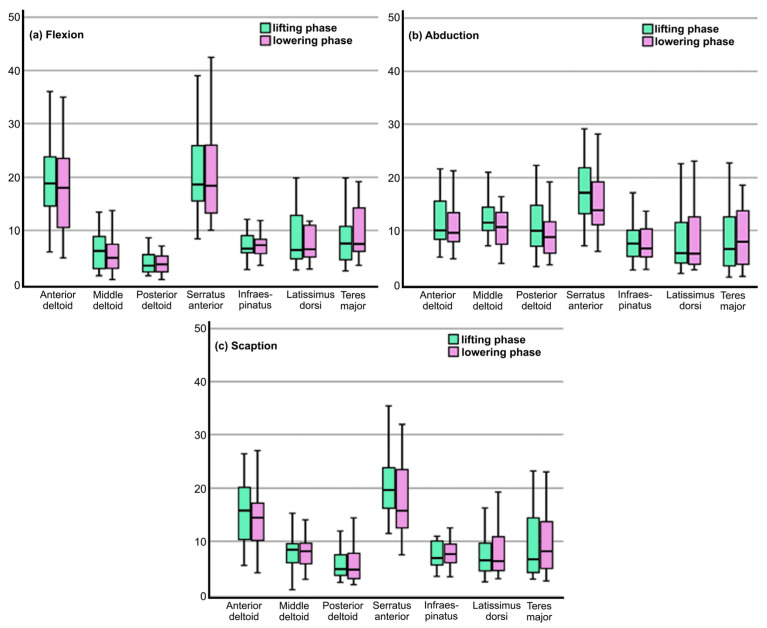
Graphic representation of the results by movement.

Table 3 shows the RMS values for all three analyzed movements during the lifting and lowering phases. These values are also presented graphically in Figure 3. Statistically significant differences were revealed only between the three parts of deltoid muscle. Post hoc analysis reveals that the anterior deltoid is statistically more active during flexion than abduction movements in both the lifting and lowering phases. The middle and posterior deltoids are statistically more active during adduction than during flexion and scaption in both phases. Statistically significant differences were not identified between flexion and scaption during either the lifting or lowering phases.

Table 4 shows the RMS values for men and women. Women exhibit greater muscle activity for all the muscles and movements analyzed, except for the middle and posterior deltoids during the lowering phase of abduction (differences −0.99 and −0.29, respectively). The infraspinatus muscle was the only one in which no statistically significant differences were observed in any of the movements studied. Significant differences were present: in the anterior deltoid during the lifting phase of abduction (*p* = 0.016, *d* = 0.19) and the lifting (*p* = 0.002, *d* = 0.29), and the lowering (*p* = 0.008, *d* = 0.22) phases of scaption; in the middle deltoid during the lowering phase of flexion (*p* = 0.027, *d* = 0.15), and the lifting phase of scaption (*p* = 0.024, *d* = 0.16); in the posterior deltoid during the lifting (*p* = 0.046, *d* = 0.13) and the lowering (*p* = 0.028, *d* = 0.15) phases of abduction, and the lifting (*p* = 0.010, *d* = 0.20) and the lowering (*p* = 0.027, *d* = 0.15) phases of scaption; in the serratus anterior during the lifting phase of abduction (*p* = 0.033, *d* = 0.17); in the latissimus dorsi during the lowering phase of scaption (*p* = 0.037, *d* = 0.14); and in the teres major during the lifting (*p* = 0.038, *d* = 0.15) and the lowering (*p* = 0.016, *d* = 0.07) phases of abduction.

## 4. Discussion

This study measures muscle activation in the shoulder region when the dominant upper limb is lifted and lowered actively without any load. The data show that there was no statistically significant difference in the activation of any of the studied muscles, whether classified as agonists or antagonists, when comparing the lifting or lowering phases. Hawkes et al. also observed the simultaneous activation of the muscles acting as agonist for the movement and their antagonistic counterparts [29]. Although these authors found multiple statistically significant differences between the lifting and lowering phases, the electromyographic signal was normalized differently; therefore, data comparison between the two studies must be approached with caution.

Although not statistically significant, small differences were observed that tended to favor concentric activation of agonist muscles over eccentric activation of antagonist muscles, a pattern that could be relevant to the direction of limb movement. This reveals a complex pattern of muscle activity coordination at each moment throughout the full range of motion [30]. Therefore, the simplistic view of muscles as either isolated motors or members of pairs of forces should be abandoned. It is preferable to think of all shoulder muscles as working together in coordinated synergy [31].

Among the muscles included in this study, the serratus anterior, considered one of the primary muscles responsible for proper scapulohumeral rhythm while stabilizing the scapula against the rib cage [32], and preventing scapular winging and anterior tilt [33], was the most active muscle in the three movements studied, both during the lifting and lowering phases, with a range between 23.27% MVIC during the lifting phase of flexion and 15.69% MVIC during the lowering phase of abduction. Adachi et al. measured the activity of the serratus anterior muscle while asymptomatic subjects performed several exercises without additional load [34]. Of the exercises analyzed by these authors, only two involve activation greater than a simple elevation: the ‘cat and dog scapular protraction exercise’ (26.7% MVIC) and the prone exercise with trunk extension and 135° upper limb elevation (25.6% MVIC), whereas the other analyzed exercises do not reach 10% MVIC [34]. Ekstrom et al. studied the activity of the serratus anterior muscle during exercises involving a 5RM load. In the scaption movement above 120° elevation, activation reached 90% MVIC, increasing to 100% MVIC in diagonal elevation. However, in horizontal extension with external rotation, activation was only 9% MVIC, regardless of the load used [35]. In summary, some of the exercises analyzed in both studies involve less serratus anterior activation, even with added load, than simple natural elevation. Exercises involving additional weights during limb elevation may therefore represent one of the most effective ways of strengthening the serratus anterior. However, it should be noted that this approach may have certain limitations in cases of shoulder pathology or scapular dyskinesia associated with subacromial pain. While some studies have suggested that serratus anterior activity is reduced in individuals with shoulder impingement compared with healthy controls, the evidence in this area remains somewhat limited [36]. Further research is needed to determine whether this weakness is a cause or consequence of the pathology.

Of the two external rotator muscles analyzed in this study, the posterior deltoid is more active during the lifting phase (5.23; 11.27 and 6.14% MVIC for flexion, abduction, and scaption, respectively) than during the lowering phase (4.43; 9.37 and 5.68% MVIC for flexion, abduction, and scaption, respectively). Conversely, the infraspinatus was less active during lifting (8.01; 7.98 and 8.62% MVIC for flexion, abduction and scaption respectively) than during lowering (8.52; 9.06 and 8.63% MVIC for flexion, abduction and scaption respectively). The infraspinatus and the teres minor muscles provide the primary external rotation force of the humeral head [37], thus facilitating limb elevation [28]. Nevertheless, the infraspinatus also plays an important role in stabilization by preventing anterior translation of the humeral head in the horizontal plane [38]. As different parts of the infraspinatus demonstrate different activation patterns [39], it is too simplistic to assign a single specific role to a muscle during movement that occurs in all three planes of space.

The role of the rotator cuff in balancing pairs of forces around the humeral head, both in the horizontal plane and in compensating for superior translation due to deltoid muscle action, thereby minimizing impingement of the subacromial structures, has been the subject of extensive research [40,41,42,43]. Nevertheless, some authors have suggested that the traditional view that normal shoulder function as being achieved through a balance between the deltoid muscle and the rotator cuff alone, disregarding the other adductor muscles, is inadequate [44]. Furthermore, many studies have found that rotator cuff injuries are prevalent in asymptomatic subjects [19,45,46] and in the contralateral asymptomatic shoulder of symptomatic subjects [47].

The activation of latissimus dorsi and teres major muscles during the lifting phase of flexion, abduction, and scaption found in this study suggest that they may play a more significant role in the normal biomechanics of the glenohumeral joint than is traditionally assumed. It appears that they can compensate for the superior translation of the humeral head during arm elevation [44], which could provide a rationale for the asymptomatic biomechanics of a shoulder with a rotator cuff tear. In particular, the teres major must be given due consideration [44]. Like the rotator cuff, it is a scapulohumeral monoarticular muscle with a moment arm greater than that of the infraspinatus or subscapularis muscles [48]. Furthermore, it is more active during the ascent than the descent of the limb when lifting loads [44].

With regard to gender, a greater degree of muscle activation was demonstrated by women than by men, with statistically significant differences observed in a number of cases, both during the lifting and lowering phases of the analyzed movements. These findings are consistent with those of Zancarano et al. who found greater muscle activation in women during isometric scaption [49]. Motabar et al. also found greater activation of the rotator cuff in women than in men during submaximal exertions [50]. In this study women demonstrated lower normalization MVIC in all the analyzed muscles (Table 1) Therefore, it can be hypothesized that woman may require a higher percentage of muscle activation than men to perform the same task.

Although electromyography is considered the gold standard for studying muscle activation, several authors have highlighted the limitations of this technique, particularly when studying the shoulder region [51]. Due to the intrinsic instability of the glenohumeral joint, muscle force is generated not only to create joint torque but also to provide joint stabilization [52]. In this study, the data on muscle activation did not conform to a normal distribution, and the high standard deviations (in some cases greater than the mean) imply a considerable degree of inter-individual variability. This is also supported by the review of Kinsella et al. who found consistent activation trends for a few muscles, but inconsistent results for most of the muscles investigated [51].

Limitations. The use of a university-based convenience sample may compromise the generalizability of the findings, given the possibility of selection bias. The small sample size increases the probability of Type II error, which could result in the inability to detect certain statistical differences. All movements were executed without external loads in a predetermined sequence; therefore, the issue of potential fatigue was not addressed. Given the substantial number of comparisons conducted in the present study, together with the relatively large standard deviations observed in the normalized EMG values, which indicate a considerable degree of inter-individual variability, there is an elevated risk of type I error; consequently, these findings ought to be interpreted with caution.

This study only analyzed the dominant arm and examined only a limited number of muscles while focusing exclusively on limb elevation in front of the coronal plane whereas daily activities involve a multitude of other shoulder movements. Furthermore, the use of a wall-guided task, although not involving force against the surface, introduced continuous tactile feedback that may have influenced shoulder muscle co-activation patterns. The observed balanced activity between agonists and antagonists might therefore reflect, at least in part, the specific guided condition rather than fully unconstrained, natural daily movements. To achieve a more accurate comparison between genders or age, a larger sample size would be required. Additionally, in order to gain a deeper understanding of the biomechanics of this region, it would be necessary to analyze the activity of a greater number of muscles simultaneously.

## 5. Conclusions

The biomechanics of the shoulder complex are characterized by a multifaceted interplay of muscle activations occurring simultaneously. In this sample, the activity exhibited by the shoulder muscles under observation was found to be comparable, with muscles functioning as either agonists or antagonists during shoulder elevation. This finding was not anticipated. Despite the exploratory nature of the present study, its findings may serve to inform future research on muscle activation in healthy shoulders during a more varied range of functional tasks.

## Figures and Tables

**Table 1:** Description of the sample.

	Total Sample Mean ± SD or *n* (%)	Men Mean ± SD	Women Mean ± SD	*p*
Gender				
Men	18 (53%)			
Woman	16 (47%)			
Age (years)	35.68 ± 12.99	31.61 ± 11.89	40.25 ± 13.00	0.070 **
Height (cm)	171.21 ± 10.24	178.33 ± 6.51	163.19 ± 7.28	0.000 *
Weight (kg)	69.59 ± 14.05	78.00 ± 7.99	60.13 ± 13.48	0.000 *
BMI	23.54 ± 3.34	24.51 ± 2.11	22.45 ± 4.13	0.072 *
Dominance				
Right	26 (76.5%)	15	11	0.276 ***
Left	8 (23.5%)	3	5	
Anterior deltoid (MVIC-µV)	807.94 ± 425.58	973.11 ± 439.24	609.74 ± 320.23	0.012 *
Middle deltoid (MVIC-µV)	1513.00 ± 747.68	1667.09 ± 839.70	1339.66 ± 608.44	0.297 **
Posterior deltoid (MVIC-µV)	752.93 ± 384.66	858.42 ± 396.79	634.25 ± 344.55	0.090 *
Serratus anterior (MVIC-µV)	334.55 ± 263.26	378.44 ± 304.00	280.54 ± 201.15	0.511 **
Infraspinatus (MVIC-µV)	469.41 ± 204.02	516.53 ± 203.70	412.87 ± 196.15	0.149 *
Latissimus dorsi (MVIC-µV)	213.13 ± 139.96	246.48 ± 131.08	175.62 ± 144.18	0.143 *
Teres major (MVIC-µV)	341.50 ± 247.79	428.72 ± 276.04	243.37 ± 171.24	0.039 **

Abbreviations: SD, standard deviation; BMI, body mass index; MVIC-µV, Maximal Voluntary Isometric Contraction expressed in microvolts; * student’s *t*; ** Mann–Whitney *U*; *** Fischer’s exact test.

**Table 2:** Normalized RMS values (%MVIC) for the lifting and lowering phases by movement.

		Lifting Mean ± SD	Lowering Mean ± SD	Mean Difference [95% CI]	*p* *	*d*	power
Flexion	Anterior deltoid	20.41 ± 8.27	17.97 ± 8.71	2.43 [−1.74; 6.61]	0.339	0.29	0.77
	Middle deltoid	7.31 ± 4.85	6.20 ± 3.33	1.11 [−0.90; 3.13]	0.320	0.27	0.72
	Posterior deltoid	5.23 ± 4.48	4.43 ± 2.96	0.81 [−1.03; 2.65]	0.589	0.21	0.79
	Serratus anterior	23.27 ± 10.90	19.82 ± 8.08	3.45 [−1.65; 8.56]	0.315	0.36	0.86
	Infraspinatus	8.01 ± 4.29	8.52 ± 4.68	−0.50 [−2.75; 1.74]	0.519	0.11	0.60
	Latissimus dorsi	12.14 ± 11.40	13.79 ± 13.24	−1.65 [−7.78; 4.48]	0.487	0.13	0.60
	Teres major	12.39 ± 12.73	12.66 ± 11.10	−0.27 [−6.24; 5.70]	0.485	0.02	0.49
Abduction	Anterior deltoid	12.74 ± 6.47	11.50 ± 5.01	1.24 [−1.65; 4.13]	0.452	0.21	0.70
	Middle deltoid	13.09 ± 5.08	11.29 ± 4.69	1.80 [−0.61; 4.21]	0.135	0.36	0.73
	Posterior deltoid	11.27 ± 4.78	9.37 ± 4.24	1.90 [−0.33; 4.12]	0.105	0.41	0.78
	Serratus anterior	18.08 ± 6.94	15.69 ± 7.09	2.40 [−1.33; 6.12]	0.138	0.34	0.69
	Infraspinatus	7.98 ± 3.90	9.06 ± 5.26	−1.07 [−3.39; 1.24]	0.638	0.23	0.84
	Latissimus dorsi	10.79 ± 10.02	10.79 ± 10.89	0.01 [−5.15; 5.22]	0.704	0.00	0.70
	Teres major	10.78 ± 11.18	10.79 ± 11.21	−0.01 [−5.60; 5.59]	0.835	0.00	0.84
Scaption	Anterior deltoid	16.41 ± 6.72	14.27 ± 6.04	2.13 [−1.03; 5.30]	0.194	0.33	0.73
	Middle deltoid	9.07 ± 4.74	8.05 ± 3.35	1.02 [−0.97; 3.00]	0.425	0.25	0.76
	Posterior deltoid	6.14 ± 3.58	5.68 ± 3.22	0.46 [−1.18; 2.11]	0.704	0.14	0.78
	Serratus anterior	20.95 ± 6.80	18.06 ± 7.36	2.89 [−0.87; 6.65]	0.125	0.40	0.79
	Infraspinatus	8.62 ± 5.14	8.63 ± 4.75	−0.01 [−2.46; 2.45]	0.753	0.00	0.75
	Latissimus dorsi	12.04 ± 13.65	11.68 ± 10.95	0.36 [−5.72; 6.45]	0.913	0.03	0.91
	Teres major	10.87 ± 9.17	12.36 ± 12.65	−1.49 [−6.93; 3.94]	0.753	0.14	0.82

Abbreviations: RMS, Root Mean Square; MVIC, Maximal Voluntary Isometric Contraction; SD, standard deviation; CI, confidence interval; * Mann–Whitney *U* test; *d*, Cohen’s *d*.

**Table 3:** Normalized RMS values (%MVIC) for movement.

		Flexion	Abduction	Scaption				*p* **	
		Mean ± SD	Mean ± SD	Mean ± SD	*p* *	η^2^	flex-abd	abd-scap	flex-scap
Lifting	Anterior deltoid	20.41 ± 8.27	12.74 ± 6.47	16.41 ± 6.72	0.000	0.164	0.000	0.065	0.265
	Middle deltoid	7.31 ± 4.85	13.09 ± 5.08	9.07 ± 4.74	0.000	0.200	0.000	0.002	0.251
	Posterior deltoid	5.23 ± 4.48	11.27 ± 4.78	6.14 ± 3.58	0.000	0.280	0.000	0.000	0.432
	Serratus anterior	23.27 ± 10.90	18.08 ± 6.94	20.95 ± 6.80	0.152	0.062	0.070	0.132	0.759
	Infraspinatus	8.01 ± 4.29	7.98 ± 3.90	8.62 ± 5.14	0.905	0.005	0.709	0.982	0.690
	Latissimus dorsi	12.14 ± 11.40	10.79 ± 10.02	12.04 ± 13.65	0.778	0.003	0.515	0.938	0.567
	Teres major	12.39 ± 12.73	10.78 ± 11.18	10.87 ± 9.17	0.633	0.004	0.341	0.580	0.687
Lowering	Anterior deltoid	17.97 ± 8.71	11.50 ± 5.01	14.27 ± 6.04	0.004	0.136	0.003	0.206	0.389
	Middle deltoid	6.20 ± 3.33	11.29 ± 4.69	8.05 ± 3.35	0.000	0.235	0.000	0.019	0.112
	Posterior deltoid	4.43 ± 2.96	9.37 ± 4.24	5.68 ± 3.22	0.000	0.268	0.000	0.001	0.301
	Serratus anterior	19.82 ± 8.08	15.69 ± 7.09	18.06 ± 7.36	0.131	0.050	0.051	0.162	0.576
	Infraspinatus	8.52 ± 4.68	9.06 ± 5.26	8.63 ± 4.75	0.960	0.002	0.784	0.833	0.950
	Latissimus dorsi	13.79 ± 13.24	10.76 ± 10.89	11.68 ± 10.95	0.193	0.012	0.071	0.440	0.297
	Teres major	12.66 ± 11.10	10.78 ± 11.21	12.36 ± 12.65	0.441	0.005	0.201	0.477	0.560

Abbreviations: RMS, Root Mean Square; MVIC, Maximal Voluntary Isometric Contraction; SD, standard deviation; * Kruskal–Wallis *H* test; η^2^ eta squared; ** post hoc Dunn test; flex, flexion; abd, abduction; scap, scaption.

**Table 4:** Normalized RMS values (%MVIC) for gender.

			Men Mean ± SD	Women Mean ± SD	Mean Difference [95% CI]	*p* *	*d*	power
Flexion	Lifting	Anterior deltoid	16.13 ± 6.90	25.54 ± 6.85	9.41 [4.51; 14.31]	0.461	0.02	0.11
		Middle deltoid	5.44 ± 3.56	9.42 ± 5.33	3.99 [0.85; 7.12]	0.107	0.84	0.36
		Posterior deltoid	4.51 ± 5.25	6.05 ± 3.41	1.55 [1.59; 4.68]	0.395	0.02	0.13
		Serratus anterior	22.62 ± 13.17	24.08 ± 7.67	1.46 [−7.02; 9.94]	0.917	0.01	0.05
		Infraspinatus	6.39 ± 3.18	10.10 ± 4.73	3.71 [0.65; 6.75]	0.059	0.12	0.48
		Latissimus dorsi	6.88 ± 5.36	18.91 ± 13.59	12.04 [3.89; 20.18]	0.121	0.08	0.34
		Teres major	9.80 ± 10.98	15.72 ± 14.41	5.92 [3.23; 15.08]	0.362	0.03	0.15
	Lowering	Anterior deltoid	14.20 ± 8.51	22.50 ± 6.71	8.30 [2.77; 13.82]	0.795	0.01	0.06
		Middle deltoid	4.89 ± 2.61	7.67 ± 3.51	2.79 [0.64; 4.93]	0.027	0.15	0.61
		Posterior deltoid	3.69 ± 2.68	5.26 ± 3.11	1.57 [−0.45; 3.59]	0.116	0.08	0.35
		Serratus anterior	19.77 ± 8.78	19.87 ± 7.56	0.10 [−6.31; 6.52]	0.746	0.01	0.06
		Infraspinatus	7.12 ± 4.16	10.31 ± 4.84	3.19 [0.06; 6.44]	0.085	0.10	0.41
		Latissimus dorsi	7.38 ± 5.83	21.48 ± 15.59	14.10 [5.14; 23.06]	0.062	0.11	0.47
		Teres major	10.42 ± 10.37	15.53 ± 11.73	5.11 [2.89; 13.10]	0.523	0.02	0.10
Abduction	Lifting	Anterior deltoid	9.85 ± 3.95	16.01 ± 7.31	6.16 [1.99; 10.33]	0.016	0.19	0.70
		Middle deltoid	12.37 ± 6.10	13.85 ± 3.77	1.48 [−2.15; 5.10]	0.075	0.11	0.43
		Posterior deltoid	11.03 ± 4.48	11.52 ± 5.23	0.49 [−2.96; 3.94]	0.046	0.13	0.52
		Serratus anterior	14.96 ± 6.26	21.93 ± 5.86	6.98 [2.31; 11.64]	0.033	0.17	0.58
		Infraspinatus	6.39 ± 3.27	9.78 ± 3.86	3.39 [0.81; 5.96]	0.065	0.12	0.46
		Latissimus dorsi	6.19 ± 5.63	15.69 ± 11.44	9.51 [2.92; 16.09]	0.076	0.10	0.43
		Teres major	9.58 ± 13.81	12.05 ± 7.72	2.47 [5.54; 10.49]	0.038	0.15	0.56
	Lowering	Anterior deltoid	9.84 ± 4.59	13.38 ± 4.93	3.54 [0.10; 6.98]	0.062	0.12	0.47
		Middle deltoid	11.77 ± 5.89	10.78 ± 3.07	−0.99 [−4.36; 2.38]	0.428	0.02	0.12
		Posterior deltoid	9.52 ± 4.24	9.22 ± 4.38	−0.29 [−3.35; 2.77]	0.028	0.15	0.61
		Serratus anterior	15.08 ± 8.18	16.40 ± 5.84	1.32 [−4.28; 6.93]	0.548	0.59	0.09
		Infraspinatus	7.07 ± 4.21	11.30 ± 5.56	4.23 [0.69; 7.76]	0.095	0.10	0.39
		Latissimus dorsi	5.97 ± 5.92	16.20 ± 12.76	10.23 [2.71; 17.75]	0.053	0.13	0.50
		Teres major	9.08 ± 12.91	12.61 ± 9.17	3.53 [4.75; 11.81]	0.016	0.07	0.29
Scaption	Lifting	Anterior deltoid	12.91 ± 5.58	20.90 ± 5.31	7.99 [4.01; 11.97]	0.002	0.29	0.91
		Middle deltoid	6.65 ± 2.78	11.79 ± 5.07	5.14 [2.18; 8.09]	0.024	0.16	0.63
		Posterior deltoid	4.62 ± 2.20	7.84 ± 4.11	3.22 [0.84; 5.60]	0.010	0.20	0.76
		Serratus anterior	19.75 ± 7.56	22.43 ± 5.67	2.68 [−2.52; 7.88]	0.460	0.02	0.11
		Infraspinatus	6.89 ± 3.98	10.70 ± 5.71	3.81 [0.36; 7.26]	0.316	0.03	0.17
		Latissimus dorsi	6.21 ± 5.41	19.04 ± 17.15	12.83 [3.08; 22.59]	0.074	0.11	0.43
		Teres major	8.68 ± 8.99	13.49 ± 8.97	4.81 [1.59; 11.21]	0.292	0.04	0.18
	Lowering	Anterior deltoid	11.48 ± 5.27	17.62 ± 5.26	6.15 [2.39; 9.90]	0.008	0.22	0.79
		Middle deltoid	6.94 ± 2.93	9.31 ± 3.44	2.37 [−0.14; 4.59]	0.063	0.11	0.46
		Posterior deltoid	4.63 ± 2.68	6.85 ± 3.45	2.21 [0.07; 4.36]	0.027	0.15	0.61
		Serratus anterior	15.87 ± 6.33	20.59 ± 7.90	4.72 [−0.81; 10.25]	0.287	0.05	0.18
		Infraspinatus	7.22 ± 4.19	10.23 ± 4.97	3.01 [0.30; 6.32]	0.126	0.08	0.33
		Latissimus dorsi	6.13 ± 5.62	17.57 ± 12.25	11.44 [4.47; 18.42]	0.037	0.14	0.56
		Teres major	8.79 ± 8.72	16.16 ± 15.18	7.37 [1.35; 16.09]	0.326	0.03	0.16

Abbreviations: RMS, Root Mean Square; MVIC, Maximal Voluntary Isometric Contraction; SD, standard deviation; CI, confidence interval; * ANCOVA adjusted for anthropometric and MVIC data; *d*, Cohen’s *d*.

## Data Availability

The research data are available on Dataverse (Havard.edu): https://doi.org/10.7910/DVN/XPMOKO.
